# Detection and Whole-Genome Characteristics of *Bordetella trematum* Isolated from Captive Snakes

**DOI:** 10.3390/pathogens14010049

**Published:** 2025-01-09

**Authors:** Magdalena Zając, Inga Bona, Magdalena Skarżyńska, Renata Kwit, Anna Lalak, Ewelina Skrzypiec, Emilia Mikos-Wojewoda, Paulina Pasim, Dominika Wojdat, Weronika Koza, Dariusz Wasyl

**Affiliations:** Department of Microbiology, National Veterinary Research Institute, 24-100 Pulawy, Poland; magdalena.skarzynska@piwet.pulawy.pl (M.S.); renata.kwit@piwet.pulawy.pl (R.K.); anna.lalak@piwet.pulawy.pl (A.L.); ewelina.skrzypiec@piwet.pulawy.pl (E.S.); emilia.mikos-wojewoda@piwet.pulawy.pl (E.M.-W.); paulina.pasim@piwet.pulawy.pl (P.P.); dominika.wojdat@piwet.pulawy.pl (D.W.); weronika.koza@piwet.pulawy.pl (W.K.); wasyl@piwet.pulawy.pl (D.W.)

**Keywords:** *Bordetella trematum*, reptile, snake, WGS, antimicrobial resistance

## Abstract

*Bordetella trematum* is a rare member of the genus *Bordetella*, primarily associated with human wound infections rather than respiratory diseases. The bacterium has been isolated from various clinical specimens, including ear inflammatory discharge, diabetic ulcers, and chronic wounds. The study aimed to characterize the genomes and antimicrobial resistance (AMR) profiles of *B. trematum* obtained from the fecal samples of asymptomatic highland eyelash pit vipers (*Bothriechis schlegelii*). The identification was conducted using MALDI-TOF mass spectrometry and biochemical tests. AMR was assessed using the microbroth dilution method, while whole-genome sequencing was performed on the Illumina NextSeq platform. The isolates displayed characteristic *B. trematum* biochemical features and demonstrated a resistance to cefotaxime, ciprofloxacin, and trimethoprim, while one also exhibited a resistance to ceftazidime. The whole-genome sequencing and comparison with limited public data revealed a high diversity within *B. trematum*, reaching >48,000 single nucleotide polymorphisms (SNPs), with 64 SNP differentiating tested snake isolates and thus, being considered epidemiologically unrelated. This is the first report of *B. trematum* isolated from an animal source in Europe. The findings provide valuable insights into this rare bacterium’s phenotypic and genomic characteristics, addressing an important knowledge gap in its ecology and AMR profile.

## 1. Introduction

*Bordetella* (*B*.) *trematum* is a Gram-negative bacillus belonging to the genus *Bordetella* in the family of *Alcaligenaceae* [1,2,3,4]. Compared to other species, the genus *B. trematum* remains rare and insufficiently characterized. Bordetellae comprise species of significant importance in human and veterinary medicine, as well as environmental bacteria [5]. Notable human pathogens include *B. pertussis*, the causative agent of whooping cough, and *B. parapertussis*, which has two lineages: one causing a milder whooping cough-like illness in humans and the other affecting sheep with respiratory symptoms [5]. *B. bronchiseptica* colonizes a wide variety of animals, including pigs, cats, and dogs, and causes atrophic rhinitis, whereas *B. avium* affects birds suffering from respiratory diseases [6]. The first described environmental species was *B. petrii* isolated from a chlorinating bioreactor enriched by river sediment [7]. Subsequently, three other environmental species, *B. muralis*, *B. tumulicola*, and *B. tumbae*, were isolated from 1300-year-old mural paintings inside a stone chamber in Japan [8].

Unlike other “classical” *Bordetella* species, *B. trematum* is not typically associated with respiratory diseases but it has been primarily linked to human wound infections. Isolations from ear inflammatory discharge, diabetic ulcers, and chronic wounds, particularly affecting the lower limbs, have been reported [1,2,4]. First described in 1996, *B. trematum* was identified as a novel species in the family *Alcaligenaceae* [1]. As an opportunistic pathogen, *B. trematum* poses a potential threat to vulnerable populations, including the elderly, infants, and immunocompromised individuals.

The bacterium is encapsulated, does not form spores, and is motile via peritrichous flagella [9]. Due to our poor knowledge of *B. trematum*, there are few reports on its biochemical characteristics [1,9]. Vandamme et al. (1996) characterized *B. trematum* as non-glucose-metabolizing, catalase-positive, and urease-negative [1]. While its ability to reduce nitrates was described as variable, lysine decarboxylation was negative. The oxidase activity was also defined as negative, which could be a biochemical feature distinguishing *B. trematum* from other *Bordetella* species [1].

So far, *B. trematum* has been mainly isolated from humans, with few reports of a potential occurrence in animals. A single case was detected in a backyard poultry environment in the USA [10]. Reptiles, more precisely snakes, might be potential reservoirs of *B. trematum* [11,12]. The bacterium was isolated from the lungs of a captive Chinese cobra (*Naja atra*) suffering from acute enteritis in Hainan, China [11]. In another study, the DNA of *B. trematum* was identified from the oral cavity of the same snake species originating from Taiwan [12]. Understanding animal reservoirs could shed light on the transmission pathways, zoonotic potential, and environmental ecology of *B. trematum*.

The aim of the study was a genomic characterization of *B. trematum* isolated from fecal samples of healthy captive snakes. To our knowledge, this is the first reported isolation of this bacteria from animal sources in Europe. The study provides important information that fills the knowledge gap regarding the phenotypic traits and genomic content of this rare bacteria.

## 2. Materials and Methods

### 2.1. Bacteria Isolation and Identification

The isolates were obtained from fecal samples collected from two asymptomatic highland eyelash-pit vipers (*Bothriechis schlegelii*) in 2021. The samples were delivered to the laboratory for monitoring purposes. The laboratory procedures included pre-enrichment in buffered peptone water (BioMaxima S.A., Lublin, Poland, 1:10 *v*/*w*; 18 ± 2 h at 37 ± 1 °C), followed by selective isolation on the chromogenic culture medium CHROMagar™ Acinetobacter (CHROMagar™, La Plaine St-Denis, France). After incubation, the growth of purple colonies, typical for *Acinetobacter* but smaller than expected, were observed. The isolates were transferred to blood agar (BioMaxima S.A., Lublin, Poland) and overnight cultures (37 ± 1 °C) were identified with Matrix-Assisted Laser Desorption Ionization-Time of Flight (MALDI-TOF) using the extraction method following the producer guidelines (Bruker Daltonics GmbH, Bremen, Germany). Scores ≥ 2.0 were considered reliable for bacterial identification to the species or genus level. The biochemical features were evaluated using the VITEK system (The Vitek 2 GN ID CARD; bioMerieux, Marcy-l’Etoile, France, Vitek 2 software version 4.7.1). The oxidase was determined with Bactident™ Oxidase test strips (N,N-dimethyl-p-phenylene diammonium dichloride and α-naphthol; Merck KGaA, Darmstadt, Germany) and Oxidase Test (N,N-dimethyl-p-phenylenediamine oxalate and α-naphthol; Merck KGaA, Darmstadt, Germany). The 3% hydrogen peroxide slide test was applied to detect the catalase.

Antimicrobial resistance testing was performed using the microbroth dilution method (Sensititre^®^, TREK Diagnostic Systems, Thermo Fisher Scientific, Waltham, MA, USA) with an EUVSEC3 MIC (minimal inhibitory concentration) panel used in the official antimicrobial resistance monitoring scheme in the EU (Table 2 of the Annex to Commission Implementing Decision (UE) 2020/1729 [13]). The panel includes 15 active compounds representing nine antimicrobial classes: aminoglycosides, beta-lactams (including carbapenems), folate-path inhibitors, glycylcyclines, macrolides, phenicols, polymyxins, tetracyclines and quinolones. *Escherichia coli* ATCC 25922 was used as a reference strain tested in parallel with the tested isolates. An inoculum was made, with a density of 0.5 McFarland in 0.9% NaCl (bioMerieux, Marcy-l’Étoile, France), from a fresh culture on blood agar (Oxoid, Hampshire, United Kingdom). Then, 10 μL was transferred to 11 mL of Cation Adjusted Mueller Hinton Broth (ThermoFisher Scientific, Waltham, MA, USA). After thorough vortexing, 50 μL of inoculum was added into each well of the plate, followed by overnight incubation (37 ± 1 °C). Since there are no interpretation criteria for *Bordetella*, the MIC values were assessed according to EUCAST guidance “When there are no breakpoints in breakpoint tables? 2024-02-29” (https://www.eucast.org/clinical_breakpoints, accessed on 4 July 2024).

### 2.2. Whole-Genome Sequencing and Bioinformatic Analysis

DNA was extracted from the overnight pure blood agar culture at 37 °C using Maxwell Rapid Sample Concentrator (RSC) cultured cells DNA Kit (Promega, Madison, WI, USA). The quantity and quality of DNA were assessed using Qubit 3.0 (Thermo Fisher Scientific) and capillary electrophoresis using Fragment Analyzer (Agilent, Santa Clara, CA, USA). DNA libraries were constructed using the KAPA HyperPlus Kit (Roche, Basel, Switzerland). Whole genome sequencing was performed on the Illumina NextSeq (MidOutput Kit 2 × 150 bp, Illumina, San Diego, CA, USA). Short reads were trimmed using fastp 0.20.0 [14]. The genome was assembled using Spades v3.15.3 (https://github.com/ablab/spades, accessed on 24 January 2022). CSI Phylogeny 1.4 (CGE) was applied, with the default settings, for phylogeny tree preparation based on single nucleotide polymorphism (SNP) [15]. The available complete sequences of *B. trematum* were downloaded from the National Center for Biotechnology Information (NCBI, https://www.ncbi.nlm.nih.gov/, accessed on 30 September 2024). The online tool iTOL v6 was applied for phylogeny tree visualization [16]. The Proksee software (https://proksee.ca/, accessed on 6 November 2024) [17] was used for the visualization of the genes, BLAST comparisons (BLAST Formatter 1.0.3), and gene annotations with Prokka 1.1.1 [18]. Resistance gene identification was performed using ResFinder [19] (threshold for identity: 60%, coverage: 40%; database version: ResFinder-2.4.0) and CARD 1.2.1 [20].

## 3. Results

### 3.1. Bacteria Identification and Antimicrobial Resistance Typing

The screening of the chromogenic medium of two samples showed the suspected, and hence, atypical growth of Acinetobacter spp., defined as very small, purple colonies. MALDI-TOF revealed that both isolates (designated as PIW211 and PIW212) did not belong to the *Acinetobacter* genus but they were perfectly identified as *B. trematum*. A repeated analysis confirmed those results. The biochemical characterization showed typical *Bordetella* genus properties like the lack of sugar fermentation and the hydrolyzing of peptides (Supplementary Table S1). Both isolates exhibited the same biochemical profiles, except for the ability of PIW211 to hydrolyze tyrosine (Supplementary Table S1). Both isolates were catalase-positive. The oxidase test revealed different results depending on the derivatives used in the test. The isolates were oxidase-negative in the test containing N,N-dimethyl-p-phenylene diammonium dichloride (Bactident™ Oxidase test strips) but oxidase-positive in the test with N,N-dimethyl-p-phenylenediamine oxalate (Oxidase Test).

**Figure 1:**
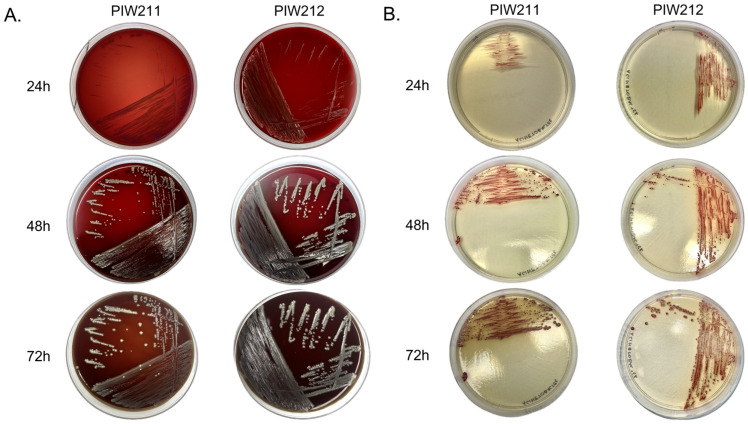
Pure cultures of *B. trematum* isolates PIW211 and PIW212 incubated for 24 h, 48 h, and 72 h in aerobic conditions on 5% horse blood agar (**A**) and CHROMagar™ Acinetobacter (**B**).

Due to the unexpected results of the MALDI-TOF evaluation, bacteria growth was evaluated on 5% horse blood agar (BioMaxima S.A., Lublin, Poland) and CHROMagar™ Acinetobacter after incubation for 24 h, 48 h, and 72 h (Figure 1A,B). The average incubation time of *B. trematum* was defined as 48–72 h for both media, but the growth on 5% horse blood agar was more abundant, compared to the CHROMagar™ Acinetobacter originally used for bacterium isolation.

The results of the antimicrobial resistance testing, detailed in Table 1, indicated that both isolates were resistant to cefotaxime, ciprofloxacin, and trimethoprim. Additionally, PIW211 was ceftazidime-resistant.

### 3.2. The Genome Analysis

The PIW211 and PIW212 genomes were 4,350,381, and 4,311,189 bp in length with GC contents of 65.45%, and 65.49%, respectively. The genomes comprised 64 and 67 contigs with N50 values of 193,049 and 278,393 bp, respectively. The PIW211 genome was estimated to contain 4036 coding sequences, whereas PIW212 had 3984. In both genomes, the cytochrome oxidase coding sequence was not detected, while both sequences contained *bvgA* and *bvgS* genes as a part of the two-component BvgA/BvgS system. No acquired resistance genes (ResFinder database) were detected in both genomes except for the *tmexD4* gene with 73.39% identity, conferring the resistance to tetracycline, doxycycline, minocycline, and tigecycline. The CARD analysis revealed the presence of the *adeF* gene (99.72% identity, 76.91% coverage), coding for the efflux pump targeting fluoroquinolones and tetracyclines, and the *fosA8* gene (96.45% identity, 54.48% coverage), which is responsible for fosfomycin resistance.

### 3.3. Phylogenetic Analysis

**Figure 2:**
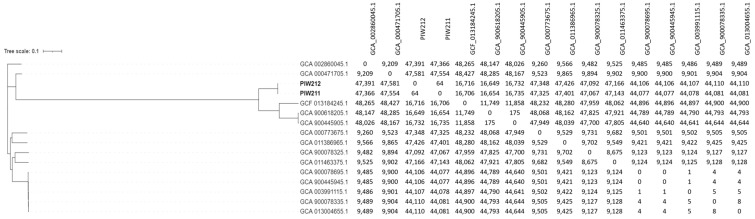
The phylogenetic tree and SNP-matrix of *B. trematum* genomes. Genomes of isolates obtained in this study were bolded.

A phylogenetic analysis of the acquired sequences with the 14 publicly available genomes (Supplementary Table S2) showed a high diversity of the strains (Figure 2). PIW211 and PIW212 exhibited low gene sequence diversity (64 SNP), but the distribution of pairwise SNP distances between all tested sequences varied from 0 SNP (GCA_900078695.1 and GCA_900445945.1) to 48,427 SNP (GCA_000471705.1 and GCF_013184245.1). The most similar genomes, GCF013184245.1, GCA900618205.1, and GCA900445905.1, were derived from humans and forest musk deer and differed more than 16,000 SNP from the studied genomes.

## 4. Discussion

### 4.1. Isolation, Identification, and Potential Reservoirs of Bordetella trematum

*B. trematum* is a relatively newly identified bacterium, and the knowledge of its occurrence in animals, the environment, or potential transmission is limited. The most relevant information refers to human infections, particularly in immunocompromised individuals or as part of mixed infections [2,4,21,22,23]. The respiratory diseases in animals are typically linked to other *Bordetella* species [6,24]. Currently, there is no documented evidence of *B. trematum* symptomatic infections in animals. Its impact or potential zoonotic link remains unclear. The occurrence of infections and the carriage of *B. trematum* in animals are not well recognized. A single case of *B. trematum* isolation from backyard flock feces and two other incidental detections in venomous snakes were documented [10,11,12].

Our study is the third description of *B. trematum* in snakes, which can indicate a specific animal reservoir. Currently, no documented cases in the literature specifically describe *B. trematum* infections linked to snakebites, which are considered a threat not only due to the cytotoxic and proteolytic effects of venom but also due to bite injury infections [25]. The microbes recovered from the bitten wound can reflect the oral flora of the biting snake [26]. Therefore, the possibility of infection with *B. trematum* in bite wounds cannot be excluded if these bacteria are present in the oral cavity of snakes [12].

One of the main challenges in assessing animals as a potential reservoir of *B. trematum* is the absence of a standardized isolation method. The known protocols recommend a Charcoal selective agar supplemented with cefalexin for *Bordetella* species and an incubation time of up to 12 days for clinical cases [27]. Due to its extended growth time, the bacterium may be missed in standard testing. Our study revealed that the best growth time for the reported *B. trematum* cultured at 37 °C varied between 48 and 72 h. It is in congruence with some reports that noted that the growth after 48 h of incubation at 37 °C further identified as *B. trematum* [28].

The other potential issue could be the discrepancies in the oxidase activity results. A few reports describe the lack of oxidase activity and indicate it as a biochemical feature distinguishing *B. trematum* from other *Bordetella* species [1,21,22]. However, oxidase-positive isolates were also reported [9,28]. Some studies link these discrepancies with different derivatives used for the oxidase tests [9]. This was also observed in the current study. The absence of the cytochrome oxidase genes in both tested *B. trematum* isolates supports Buechler’s hypothesis that the type of derivative influences the results of the oxidase reaction and might lead to false-positive observations [9]. A further analysis on a larger set of strains is necessary, as this matter may encounter conflicting information, which challenges the reliability of oxidase tests for biochemical differentiation.

### 4.2. Antimicrobial Resistance of Bordetella trematum

Although *B. trematum* is considered a relatively rare opportunistic pathogen, it has been documented to show resistance to multiple classes of antimicrobials. The most often noted resistance is the resistance to beta-lactams, including cephalosporines [9,22,23]. In some reports, *B. trematum* also showed a resistance to fluoroquinolones like ciprofloxacin, commonly used for treating various infections [22]. A case of resistance to trimethoprim and sulfamethoxazole confirmed by the detection of the *sul2* gene on the genome was previously documented [9]. The currently described resistance to cefotaxime, ciprofloxacin, and trimethoprim was partly explained by the presence of genes coding for efflux pumps targeting tetracyclines and fluoroquinolones. Unfortunately, none of the currently known resistance determinants could justify the observed resistance to cefotaxime and trimethoprim in both, and additionally, ceftazidime in one isolate. In some reports, the lack of a genetic background, despite the presence of a cephalosporin-resistance phenotype, has also been noted [23]. In the literature, the determinants of beta-lactam resistance belonging to *bla*_TEM_ or *bla*_OXA_ were reported in some species of the *Bordetella* genus [29,30]. We cannot exclude, due to the still scarce knowledge on *B. trematum* and imperfect AMR gene databases, the presence of resistance genes in the studied genomes. Simultaneously, there is limited data on the interpretation criteria for many antimicrobials for *B. trematum*, and the application of breakpoints for Enterobacterales was recommended in this case. Hence, we consider the missing MIC interpretation as a limitation of this study.

### 4.3. Invasiveness of Bordetella

The zoonotic potential of *B. trematum* is not well established. However, given that some *Bordetella* species, like *B. bronchiseptica*, are known to infect a variety of animal species (e.g., dogs, cats, pigs), there is a possibility that *B. trematum* could similarly infect animals under certain conditions. In many facultative pathogens encountering different environmental conditions during their life cycle, the two-component system (TCS) plays a dominant role in the expression of their virulence [31]. This system is known in the Bordatellae, including environmental species like *B. petrii* but not *B. homelesi* [32]. In *B. pertussis*, human respiratory infections are regulated by the BvgAS two-component system, while in *B. bronchiseptica*, the *bvg*-activated genes are similarly crucial for colonizing the animal respiratory tract [33]. The genes coding this system were identified in both of the studied *B. trematum* isolates. Their expression exceeds the current report and might trigger further in vitro and in vivo experiments.

The large-scale studies on the genomic diversity of *B. trematum* are currently also lacking. The phylogenetic analysis reported here revealed a wide diversity of several available genomes. The described strains of snake origin exhibited little genetic diversity and were not related to both human and animal ones. An SNP analysis has shown that isolates may differ by only a few SNPs, which was noticed in some human cases, particularly originating from similar ecological or geographic backgrounds. A small number of genomes limits the understanding of the population structure of *B. trematum.* Studies involving more isolates from diverse hosts and environments would provide deeper insights into its genetic variability and evolutionary trends.

## 5. Conclusions

This study contributes to knowledge-building on the presence and potential reservoirs of *B. trematum.* An investigation of the bacterium’s ecology and pathogenicity mechanisms could reveal more about its relation to animals. Due to the absence of a selective isolation method, possibly also resulting in the low occurrence of *B. trematum*, further research exploring *B. trematum* in animal hosts would be challenging. Nevertheless, our study provides an important contribution to expanding the knowledge on *B. trematum*, considering both phenotype and genotype characteristics. These findings indicate areas for further research that contribute to a more detailed understanding of the genetic diversity of this bacterium, including the genetic background of its antimicrobial resistance, especially to beta-lactams.

## Figures and Tables

**Table 1:** Minimum inhibitory concentration (MIC) values (mg/L) for the *B. trematum* strains isolated in the study. Results were interpreted according to EUCAST guidelines.

Antimicrobial	PIW211	Interpretation	PIW212	Interpretation
Amikacin	8	NA	8	NA
Ampicillin	2	S	2	S
Azithromycin	≤2	NA	≤2	NA
Cefotaxime	>4	R	>4	R
Ceftazidime	8	R	4	S
Chloramphenicol	≤8	NA	≤8	NA
Ciprofloxacin	1	R	1	R
Colistin	≤1	NA	≤1	NA
Gentamicin	2	NA	4	NA
Meropenem	≤0.03	S	≤0.03	S
Nalidixic Acid	8	NA	8	NA
Sulphamethoxazole	≤8	NA	≤8	NA
Tetracycline	≤2	S	≤2	S
Tigecycline	≤0.25	NA	≤0.25	NA
Trimethoprim	16	R	16	R

NA—not interpreted due to missing interpretation criteria, S—sensitive, R—resistant.

## Data Availability

The original contributions presented in the study are included in the article, and further inquiries can be directed to the corresponding authors. The complete genomes of *Bordetella trematum* were assigned the GenBank project number PRJNA1182378.

## Supplementary Materials

The following supporting information can be downloaded at: https://www.mdpi.com/article/10.3390/pathogens14010049/s1, Table S1: Biochemical characteristics of *B. trematum* isolates on VITEK System; Table S2: Metadata of genomes downloaded from the NCBI database and used for comparison in this study.
