# Supplementary material for: Detection and Whole-Genome Characteristics of Bordetella trematum Isolated from Captive Snakes
Source: Pathogens. 2025 Jan 9;14(1):49. doi: 10.3390/pathogens14010049 (PMC11768120; doi:10.3390/pathogens14010049)
Supplement: Supplementary file 1 [file pathogens-14-00049-s001.zip › Supplementary Tables.pdf]

Supplementary Materials

Table S1. Biochemical characteristics of *B. trematum* isolates on VITEK System.

	Abbreviation	Test (quantity/well)	Results	
			PIW211	PIW212
1.	ArgA	Arginine arylamidase (0.0324 mg/well)	-	-
2.	GGT	Gamma-glutamyltransferase (0.0228 mg/well)	-	-
3.	LysA	L-Lysine arylamidase (0.0228 mg/well)	-	-
4.	dGAL	D-galactose (0.3 mg/well)	-	-
5.	LeuA	Leucine arylamidase (0.023 mg/well)	+	+
6.	ELLM	ELLMAN's reagent (0.03 mg/well)	+	+
7.	PheA	Phenylalanine arylamidase (0.026 mg/well)	+	+
8.	ProA	L-proline arylamidase (0.023 mg/well)	+	+
9.	PyrA	L-pyrrolidonyl arylamidase (0.018 mg/well)	-	-
10.	TyrA	Tyrosine arylamidase (0.0279 mg/well)	(+)	-
11.	APPA	Ala-Phe-Pro arylamidase (0.038 mg/well)	-	-
12.	dGLU	D-glucose (0.3 mg/well)	-	-
13.	GLYG	Glycogen (0.18 mg/well)	-	-
14.	dMNE	D- mannose (0.3 mg/well)	-	-
15.	dMAL	D- maltose (0.3 mg/well)	-	-
16.	SAC	Saccharose/cane sugar (0.3 mg/well)	-	-
17.	NAG	N-acetyl-D-glucosamine (0.3 mg/well)	-	-
18.	URE	Urease (0.15 mg/well)	-	-
19.	BGALi	Indoxyl beta-galactopyranosidase (0.006 mg/well)	-	-
20.	ODC	Ornithine decarboxylase (0.15 mg/well)	+	+
21.	AARA	Alpha-arabinosidase (0.0324 mg/well)	-	-
22.	PVATE	Pyruvate (0.15 mg/well)	+	+
23.	PHC	Phosphorylcholine (0.0366 mg/well)	-	-
24.	dMLT	D-malate (0.15 mg/well)	+	+
25.	MTE	MALTOTRIOSE (0.3 mg/well)	-	-
26.	IGLM	L-glutamine (0.15 mg/well)	+	+
27.	PHOS	phosphatase (0.05 mg)	-	-
28.	dRIB2	D-Ribose 2 (0.3 mg/well)	-	-
29.	OPS	Phenylphosphonate (0.024 mg)	-	-
30.	dXYL	D-XYLOSE (0.3 mg)	-	-
31.	ADO	Adonitol (0.1875 mg/well)	-	-
32.	IARL	L-arabitol (0.3 mg/well)	-	-
33.	dCEL	D-cellobiose (0.3 mg/well)	-	-
34.	BGAL	Beta-galactosidase (0.036 mg/well)	-	-
35.	H2S	H_2S production (0.0024 mg/well)	-	-
36.	BNAG	Beta-n-acetylglucosaminidase (0.0408 mg/well)	-	-
37.	AGLTp	Glutamyl arylamidase pNA (0.0324 mg/well)	-	-
38.	GGT	Gamma-glutamyltransferase (0.0228 mg/well)	-	-
39.	OFF	Fermentation/glucose (0.45 mg/well)	-	-
40.	BGLU	Beta-glucosidase (0.036 mg/well)	-	-
41.	dMAN	D-mannose (0.3 mg/well)	-	-
42.	BXYL	Beta-xylosidase (0.0324 mg/well)	-	-
43.	BAlap	BETA-alanine arylamidase pNA (0.0174 mg/well)	-	-
44.	LIP	Lipase (0.0192 mg/well)	-	-
45.	PLE	Palatinosis (0.3 mg/well)	-	-
46.	dSOR	D-sorbitol (0.1875 mg/well)	-	-
47.	dTAG	D-tagatose (0.3 mg/well)	-	-
48.	dTRE	D-trehalose (0.3 mg/well)	-	-
49.	CIT	Citrate (sodium) (0.054 mg/well)	+	+
50.	MNT	Malonian (0.15 mg/well)	-	-
51.	5KG	5-keto-D-gluconate (0.3 mg/well)	-	-
52.	ILATk	Alkalization of L-lactate (0.15 mg/well)	+	+
53.	AGLU	Alpha-glucosidase (0.036 mg/well)	-	-
54.	SUCT	Alkalization of succinate (0.15 mg/well)	+	+
55.	NAGA	Beta-N-acetylgalactosaminidase (0.0306 mg/well)	-	-
56.	AGAL	Alpha-galactosidase (0.036 mg/well)	-	-

57.	PHOS	Phosphatase (0.0504 mg/well)	-	-
58.	GlyA	Glycine arylamidase (0.012 mg/well)	-	-
59.	ODC	Ornithine decarboxylase (0.3 mg/well)	-	-
60.	LDC	Lysine decarboxylase (0.15 mg/well)	-	-
61.	lHISa	L-histidine absorption (0.087 mg/well)	-	-
62.	CMT	Coumaran (0.126 mg/well)	-	-
63.	BGUR	Beta-glucoronidase (0.0378 mg/well)	-	-
64.	0129R	Resistance to O/129 (vibriostatic agent) (0.0105 mg/well)	-	-
65.	GGAA	Glu-Gly-Arg arylamidase (0.0576 mg/well)	-	-
66.	IMLTa	L-malate absorption (0.042 mg/well)	-	-
67.	ILATa	L-lactate absorption (0.186 mg/well)	-	-

Table S2. Metadata of genomes downloaded from the NCBI database and used for comparison in this study.

No.	ID	GenBank Number	NCBI RefSeq Number	Species	Host	Source	Geographic location	Release year
**1**	Btrematum1.0	GCA_000471705.1	GCF_000471705.1	*Bordetella trematum*	No data	No data	Canada	2013
**2**	ASM77367v1	GCA_000773675.1	GCF_000773675.1	*Bordetella trematum*	*Bos taurus coreanae*	rumen	South Korea	2014
**3**	H044680328	GCA_900078695.1	GCF_900078695.1	*Bordetella trematum*	No data	No data	United Kingdom	2016
**4**	7642_7#32	GCA_900078325.1	GCF_900078325.1	*Bordetella trematum*	No data	obscured	United Kingdom	2016
**5**	7642_7#39	GCA_900078335.1	GCF_900078335.1	*Bordetella trematum*	No data	No data	South Korea	2016
**6**	ASM399111v1	GCA_003991115.1	GCF_003991115.1	*Bordetella trematum*	*Homo sapiens*	No data	No data	2019
**7**	ASM286004v1	GCA_002860045.1	GCF_002860045.1	*Bordetella trematum*	*Homo sapiens*	wound exudate	USA	2018
**8**	NCTC13366	GCA_900618205.1	GCF_900618205.1	*Bordetella trematum*	*Homo sapiens*	No data	United Kingdom	2018
**9**	52067_A01	GCA_900445945.1	GCF_900445945.1	*Bordetella trematum*	*Homo sapiens*	No data	Germany	2018
**10**	59000_B01	GCA_900445905.1	GCF_900445905.1	*Bordetella trematum*	*Homo sapiens*	leg wound	No data	2018
**11**	ASM1146337v1	GCA_011463375.1	GCF_011463375.1	*Bordetella trematum*	*Homo sapiens*	necrotic ulcer	Brazil	2020
**12**	ASM1318424v1	GCA_013184245.1	GCF_013184245.1	*Bordetella trematum*	Forest musk deer (Moschus berezovskii)	No data	China	2020
**13**	ASM1138696v1	GCA_011386965.1	GCF_011386965.1	*Bordetella trematum*	*Homo sapiens*	No data	China	2020
**14**	ASM1300465v1	GCA_013004655.1	GCF_013004655.1	*Bordetella trematum*	*Homo sapiens*	ear swab	Germany	2020
**15**	PIW211	SAMN44594626	JBJCJK000000000	*Bordetella trematum*	Eyelash-pit viper *(Bothriechis schlegelii)*	*feces*	Poland	2021
**16**	PIW212	SAMN44594627	JBJCJJ000000000	*Bordetella trematum*	Eyelash-pit viper *(Bothriechis schlegelii)*	*feces*	Poland	2021
